# Resource use by individual *Drosophila suzukii* reveals a flexible preference for oviposition into healthy fruits

**DOI:** 10.1038/s41598-020-59595-y

**Published:** 2020-02-21

**Authors:** Renate Kienzle, Lara B. Groß, Shelby Caughman, Marko Rohlfs

**Affiliations:** Institute of Ecology, Population and Evolutionary Ecology Group, University of Bremen, Bremen, Germany

**Keywords:** Agroecology, Behavioural ecology, Evolutionary ecology

## Abstract

The invasive pest fruit fly *Drosophila suzukii* is thought to be a specialist on healthy, i.e. unwounded, non-fermenting fruits. Morphological (sharp ovipositor) and neurophysiological/behavioural adaptations have been suggested to contribute to distinct adult feeding (wounded/microbe-laden fruits) and reproductive (healthy fruits) sites. We tested whether (1) variation in the overall availability of fruits, (2) variation in fruit type (healthy, wounded, fermenting), and (3) the relative abundance of different fruit types are ecological determinants of *D. suzukii* egg-laying decisions. Even though individual flies reduced their reproductive output when resource availability (blueberries) was low, a significantly higher proportion of eggs was allocated to healthy fruits, relative to wounded and fermenting fruits. However, the preference for healthy over wounded fruits declined continuously with a decrease in the relative abundance of healthy fruits and the overall reproductive output did not change. Under laboratory conditions, *D. suzukii* larvae achieved a higher developmental success on wounded than on healthy blueberries, but suffered less from density-dependent competition in healthy fruits. These data suggest that *D. suzukii*, despite showing an egg-laying preference for healthy fruits, also uses wounded/fermenting fruits as egg-laying sites, and that it may thrive well in windfall fruits.

## Introduction

*Drosophila suzukii*, a devastating invasive insect in European and American fruit plantations, has been described as being ‘…extremely fond of otherwise undamaged, ripening fruits…’^1^. Indeed, unlike most other drosophilid fruit fly species that are able to use only fruits with cracked skins for egg-laying, a serrated ovipositor enables *D. suzukii* to pierce the intact skin of many cultured and wild fruits and to insert their eggs into the fresh fruit flesh^2^. The subsequent larval development and microbial growth cause a rapid fruit decay that renders cherries, blue-, straw-, and blackberries etc. unmarketable. Recently, Karageorgi *et al*.^3^ showed that an egg-laying preference of *D. suzukii* for intact ripening over rotting strawberries is mediated by a combination of chemo- and mechanosensation. This apparent preference has been suggested to have resulted in distinct adult feeding and breeding sites^4^. Studies designed for describing neurophysiologically ‘hard-wired’ preferences, however, often overlook the impact of variation in experience and internal status, i.e. optimal foraging decisions^5^, as well as neural limitations in detecting the preferred host in naturally complex ecological settings^6^.

Conclusions regarding such ‘hard-wired’ preferences are particularly problematic for polyphagous insects^7^ – like *D. suzukii* – where individuals are confronted with hosts that vary widely in suitability and availability. For example, the extent to which a particular host is used for oviposition may be affected by the type and density of alternative hosts in the vicinity, resulting in apparently suboptimal host use^8^. Suboptimal host use may be due to limitations in the ability to perceive and integrate stimuli of a focal host, which constrain insects in making the ‘right’ and expected host choice^9,10^. Additionally, insect host choice decisions often depend on host availability, which contributes to the probability of whether female insects become egg- or time-limited^11^. Egg limitation, i.e. when the availability of preferred hosts is not limited, tends to favour specialization on a particular host, whereas increasing time limitation, i.e. when the preferred hosts are scarce and females have more eggs than they can lay and hence suffer from ‘oviposition pressure’, leads to generalist oviposition into many different hosts^12^.

In the light of this complexity of insect host plant selection^13^, we firstly tested the null hypothesis that *D. suzukii* females show no preference for oviposition into healthy fruits when presented alongside wounded and fermenting fruits. We also addressed the possibility that the individual egg-laying decisions of *D. suzukii* females depend on variation in resource availability, i.e. fruit density. We had two alternative hypotheses regarding the influence of an increase in fruit density. The preference for healthy fruits may decrease with increasing fruit density as the ability to detect distinct patch boundaries may be reduced at high densities and thus females are less able to distinguish between healthy, wounded and fermenting fruits. In consequence, at higher fruit densities we expected females to oviposit more often into the presumably non-preferred decay stages (wounded/fermenting fruits), which leads to a random distribution of eggs across all fruit categories. Alternatively, transient time- and egg-limitation under low and high resource availability, respectively, may be more relevant in *D. suzukii* oviposition decisions. That is, if confusion due to high fruit densities has an only minor influence, individuals should specialize on the preferred fruit-stage (here healthy fruits) when resources are abundant and should be less choosy when overall resource availability is low. In a second experiment, we investigated the effect of changes in the relative abundance of fruit stages on the allocation of eggs to the presumably preferred host category. As null hypothesis we expected flies to allocate their eggs proportional to the availability of different fruit categories, whereas deviations from such a proportionality would indicate some degree of specialization on either of the fruit categories offered. Experiments were carried out under controlled laboratory conditions by analysing egg-distribution patterns on blueberries that individual *D. suzukii* females produced during one egg-laying period. In a third experiment we examined potential fitness consequences of an oviposition preference, and provided a first test of the developmental success of *D. suzukii* larvae in healthy and wounded blueberries.

## Results

### Experiment 1 – *Drosophila suzukii* egg distributions as a function of variation in total fruit availability

**Figure 1:**
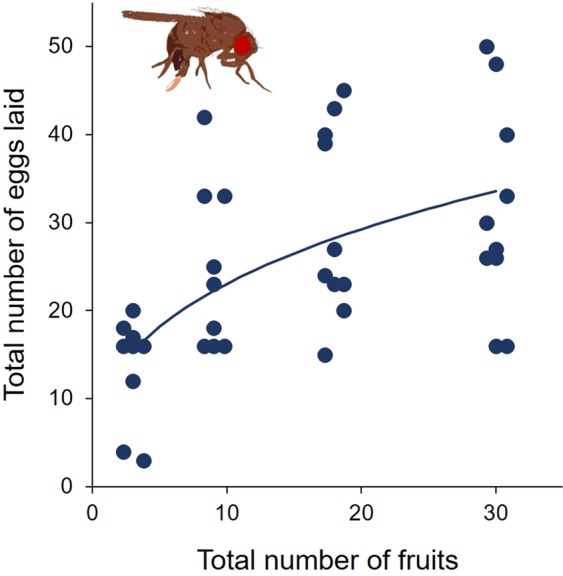
Experiment 1 – Total number of eggs laid per female as a function of total resource availability, i.e. in total 3, 9, 18 or 30 fruits. The line depicts the results of the generalised linear regression model (see Supplementary Table S1). For better visibility data are staggered around the actual number of fruits.

**Figure 2:**
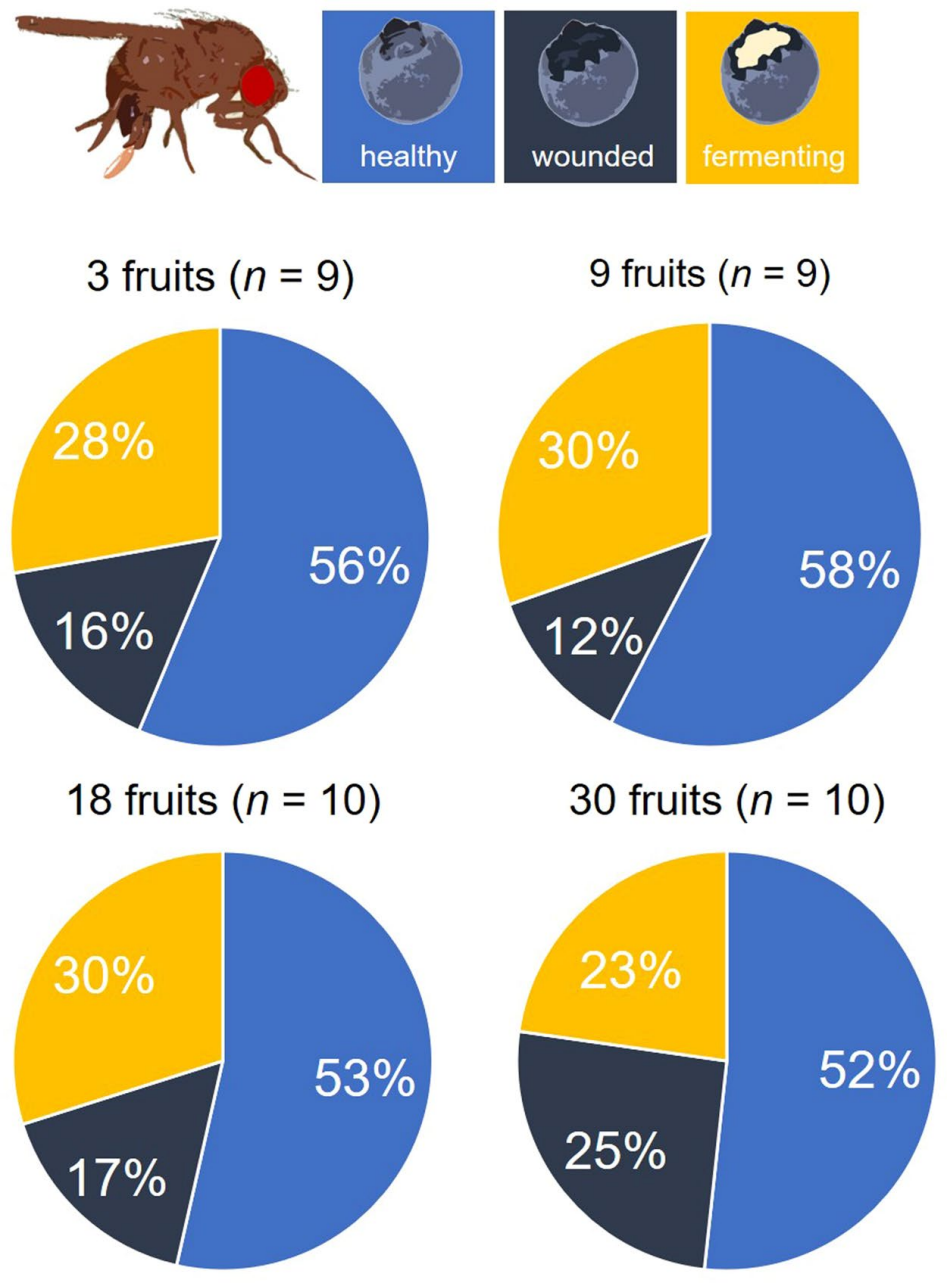
Experiment 1 – Proportion of eggs allocated to healthy (blue), wounded (dark blue) and fermenting (yellow) fruits, as a function of resource availability, i.e. in total 3, 9, 18 or 30 host fruits. The proportion of eggs deposited differed significantly between wounded and fermenting fruits (Wilcoxon signed rank test on arcsine square-root-transformed proportions; *p* = 0.0102).

When offered equal numbers of healthy, wounded and fermenting fruits for oviposition, overall, females laid significantly more eggs when total fruit availability increased (*p* < 0.001, Fig. 1, see Supplementary Table S1 for more information on the *GLM*). All fruit categories – healthy, wounded, and fermenting – were accepted as oviposition sites by *D. suzukii* (Fig. 2). The proportion of eggs individual *D. suzukii* females laid into healthy blueberries did not depend on resource availability (Type II ANOVA, *p* = 0.470) nor was it influenced by the total number of eggs the females deposited (Type II ANOVA, *p* = 0.745) (see Supplementary Table S2 for more information on the *GLM*). The overall proportion of eggs laid into healthy fruits was 0.565 ± 0.381 *s.e*., that is *D. suzukii* females laid a significantly higher proportion of eggs into healthy fruits than the 1/3 ratio expected under the null hypothesis (*GLM* estimate: 0.867 ± 0.162 *s*.*e*., *t*-value: 5.363, *p* < 0.001), whereas wounded fruits received the significantly lowest proportion of eggs, and hence seem to be the least preferred oviposition sites (Fig. 2).

**Figure 3:**
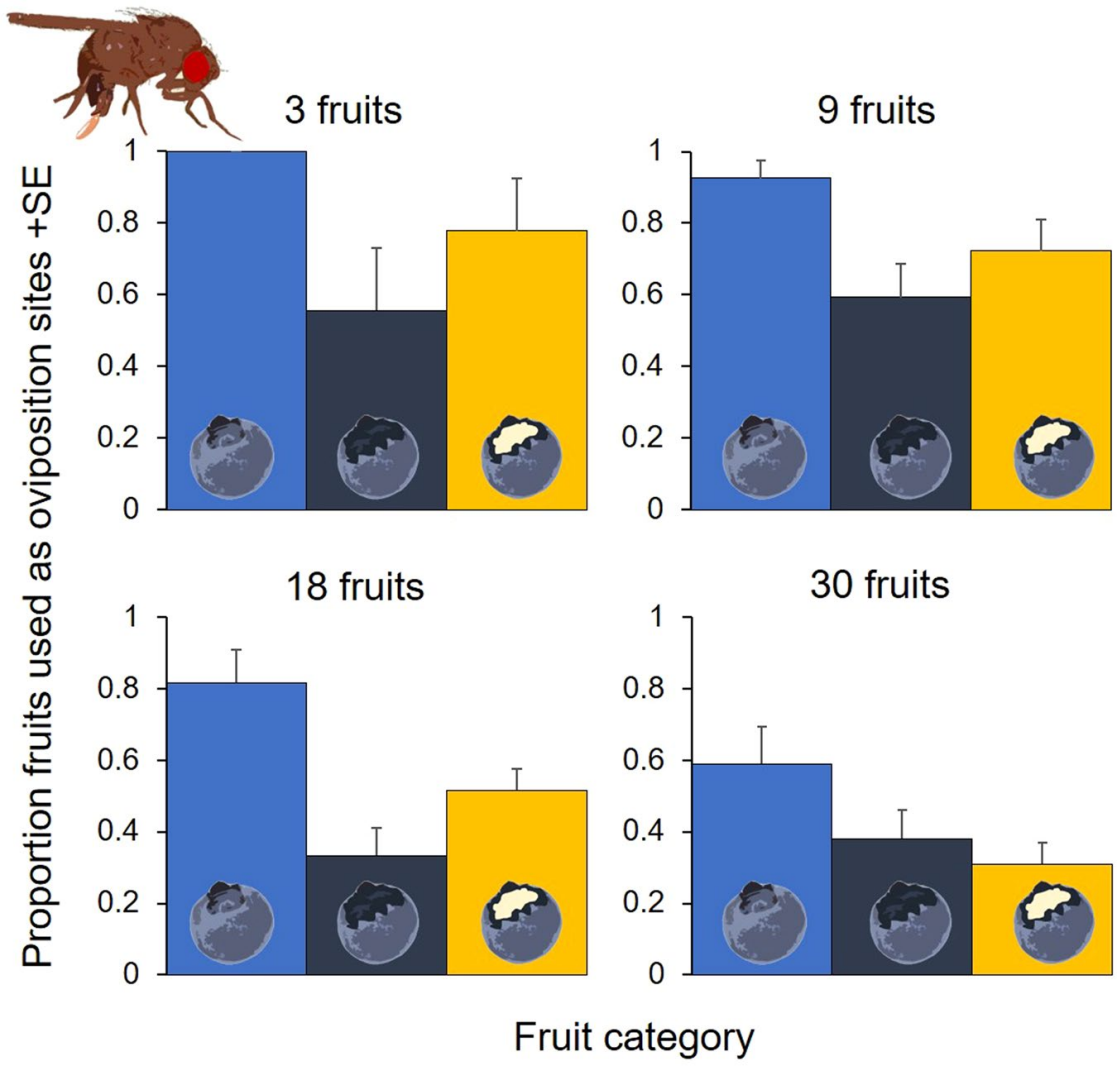
Experiment 1 – Proportion fruits used as oviposition sites, independent of the total number of eggs deposited per fruits, as a function of healthy (blue), wounded (dark blue) and fermenting (yellow) fruits, and resource availability, i.e. in total 3, 9, 18 or 30 host fruits. (see Supplementary Tables S3 and S4 for more information).

The proportion of blueberries used as oviposition sites – independent of the number of eggs laid per fruit – was affected significantly by the total resource availability (Type II ANOVA, *p* < 0.001) and the fruit status (Type II ANOVA, *p* < 0.001); also, the statistical interaction between resource availability and fruit status is significant (Type II ANOVA, *p* = 0.008) (see Supplementary Table S3 for more information on the generalised linear mixed effect model), which indicates that changes in fruit use with increasing resource availability differed among the status of the blueberries: while the proportion of host fruits used for oviposition declined significantly with increasing resource availability in healthy (*p* = 0.007) and fermenting (*p* < 0.001) blueberries, it did less so in wounded blueberries (*p* = 0.191) (Fig. 3; see Supplementary Table S4 for more information on the *GLM*s).

### Experiment 2 – *Drosophila suzukii* egg distributions as a function of variation in the proportional abundance of healthy fruits

**Figure 4:**
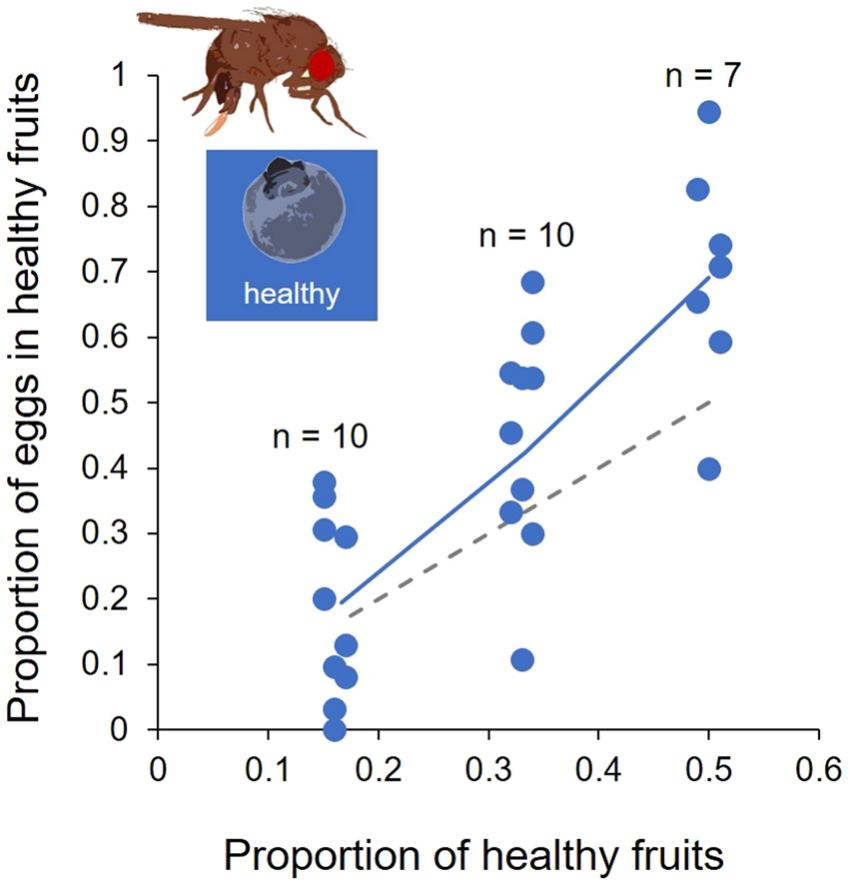
Experiment 2 – Proportions of eggs allocated to healthy fruits as a function of different proportions of healthy and wounded fruits but otherwise constant total fruit availability. The dashed grey line depicts the average allocation of eggs under the null hypothesis, the blue line the results of logistic generalised linear regression model. For better visibility data are staggered around the actual number of fruits.

To test how robust the preference for healthy fruits is observed in Experiment 1, we reduced the relative abundance of healthy versus wounded fruits from 6/6 to 4/8 and 2/10 and quantified the proportional distribution of eggs across the two fruit categories. A significantly higher proportion of eggs (~69%, *GLM* estimate: 0.716 ± 0.257, *t*-value: 2.784, *p* = 0.032) were laid into healthy fruits than expected under the null hypothesis (*H*_0_), namely 1/2 = 50%, when flies were offered an equal proportion of six healthy and six wounded fruits (6/6) (Fig. 4). This preference for healthy fruits declined with a decrease in the relative availability of healthy blueberries, 4/8: ~45% (*H*_0_: 1/3 ≈ 33.3%), *GLM* estimate: 0.416 ± 0.232, *t*-value: 1.79, *p* = 0.106; 2/10: ~18% (*H*_0_: 1/6 ≈ 16.7%), *GLM* estimate: 0.165 ± 0.290, *t*-value: 0.567, *p* = 0.584) (Fig. 4). On average, individual *D. suzukii* females laid 28.42 ± 9.78 s.d. eggs per experimental arena, which was not affected by the relative abundance of healthy fruits (*GLM* estimate: −0.154 ± 0.5167, *t*-value: −0.61, *p* = 0.547; family: *poisson*, link: *log*).

### Experiment 3 – Effect of host fruit status and intraspecific competition on the developmental success of *Drosophila suzukii* larvae

**Figure 5:**
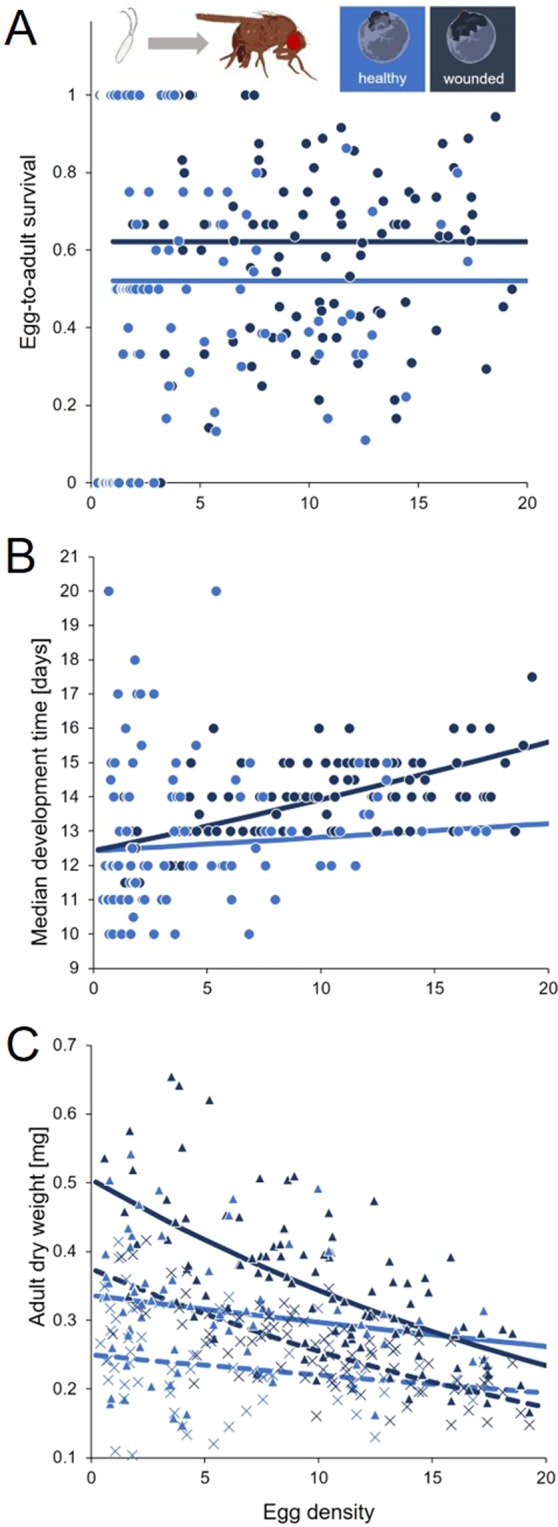
Experiment 3 – Egg-to-adult survival (**A**), development time (**B**) and adult dry weight (**C**) of *D. suzukii* that developed at different densities (eggs per mg blueberry) in healthy (blue, *n* = 111) or wounded (dark blue, *n* = 111) blue berries. Lines depict the most parsimonious *GLM* regression models (see Supplementary Tables S6 and S7). In C, triangles and crosses represent female and male body weight, respectively. Dashed lines depict regression models for male weight.

We recorded egg-to-adult development in the least and most preferred fruits catergories, that is wounded and healthy blueberries, to test whether variation in the status of potential breeding sites vary in their influence on insect fitness. Eggs deposited into wounded fruits had an approximately 10% higher probability of developing into adults (0.62 ± 0.02 s.e.) than eggs in healthy fruits (0.52 ± 0.03 s.e., *p* = 0.005), while the initial egg density hardly affected survivorship (*p* = 0.186, Fig. 5, Supplementary Tables S6 and S7 for more information on the *GLM*s).

Overall, developmental time clearly increased with initial egg density (*p* < 0.001, Fig. 5, Supplementary Tables S6 and S7). As indicated by the statistically significant interaction between fruit category and egg density (*p* = 0.034), this increase was stronger in wounded blueberries, leading to an average developmental delay of two days at high densities as opposed to only half a day in *D. suzukii* on intact fruits (Fig. 5, Supplementary Tables S6 and S7). Adult *D. suzukii* weight generally decreased with increasing egg density (*p* < 0.001, Fig. 5, Supplementary Tables S6 and S7). However, as indicated by the statistically significant interaction between fruit category and egg density (*p* < 0.001, Supplementary Tables S6 and S7), the decrease in the weight of adults emerging from the lowest to the highest density was more pronounced in wounded fruits (~41% decrease) than in intact fruits (~16% decrease). Consequently, flies emerging from wounded fruits at low densities of 1 egg per gram fruit were 1.5 times heavier than those from intact fruits, whereas flies from both fruit treatments attained the same weight at higher densities (at 15 eggs per gram: male weight ~0.21 mg, female weight ~0.28 mg). Females were generally heavier than males (p < 0.001, Supplementary Tables S6 and S7), but the sex-specific differences in body weight were not affected by egg density nor fruit status, i.e. there was no statistical interaction between these factors.

## Discussion

By combining variation in quality with availability of fruits, our results provide evidence of an oviposition preference of individual *D. suzukii* females for healthy fruits under controlled laboratory conditions. Even under strong resource limitation females did not tend to use wounded or fermenting fruits more intensively as egg-laying sites compared to conditions when the overall fruit availability was ten times higher (Experiment 1). Yet flies responded to the lower resource availability by reducing the total number of deposited eggs. This may indicate that flies experiencing resource limitation were not suffering from strong (transient) time limitation (even though they had been previously kept under strong competition for egg-laying sites, see Methods). Resource limitation therefore did not fundamentally alter the flies’ preference for healthy fruits. Compared to other drosophilids, such as *D. melanogaster*, modifications in the mechanosensory modalities determining the choice of oviposition sites may have contributed to the evolution of this predilection of *D. suzukii* for the properties of intact fruit skin^3^. However, we clearly show that this preference vanishes in response to a proportional decrease in the availability of healthy fruits (Experiment 2). In this experiment, the flies’ reproductive output remained unaffected even when the relative abundance of healthy fruits drops below a threshold where the actual preference could not be observed anymore. Therefore, it is the overall variation in all possible fruit categories that seems to regulate offspring production of *D. suzukii* rather than the absolute abundance of the otherwise preferred healthy host fruits.

At least under our experimental conditions, an overall increase in resource density did not seem to affect individual *D. suzukii* females in allocating a fairly constant 50–60% of their deposited eggs to healthy fruits. Moreover, flies flexibly responded to an increase in resource density by reducing the proportion of fruits used for oviposition in healthy and fermenting fruits. Because no such relationship was found in wounded fruits, we assume that the factors affecting the use of the latter fruit category differ from those determining egg-laying decisions in the presence of the other two fruit categories. To test this assumption, we need detailed behavioural observations quantifying correlations between the likelihood of visiting a specific fruit category, patch residence times, feeding and clutch size decisions.

The sharpened and strongly serrated ovipositor of *D. suzukii* has evolved from ancestors with only weakly serrated and sclerotized shovel-like valves^14^. As proposed by Karageorgi *et al*.^3^, avoidance of egg-laying into smooth and slushy fruit flesh coupled with this morphological prerequisite to pierce intact fruit skin seem to have resulted in the evolution of a drosophilid lineage capable of exploiting a resource that is inaccessible to most of their phylogenetic relatives. Unlike more distantly related Drosophilidae that have evolved into real herbivores and lost their ability to detect yeast-born volatile metabolites^15^, *D. suzukii* is attracted by such olfactory cues^16^. Yeast fungi likely play a role in supporting larval development^17^, and fermenting, i.e. yeast-laden, fruits may be an important proteinaceous food source for egg-maturating females^4^. An even stronger egg-laying preference for healthy fruits may thus be limited by the comparatively close relatedness to species whose reproductive strategies have evolved in the context of wounded and fermenting fruits, and/or the need to use fermenting fruits as essential adult feeding sites. Therefore, reproductive and feeding sites seem not as clearly separated as it has been proposed by other studies^4^. Based on the results of Experiment 2 where the preference for healthy fruits steadily declined with the relative increase of wounded fruits, we propose that trade-offs between feeding and the probability of finding healthy fruits for oviposition or the complete lack of those host fruits may make *D. suzukii* ‘switch’ to fermenting or wounded fruits exclusively, without reducing the overall reproductive output. Moreover, we observed ample intra-individual variation in oviposition site selection, which additionally weakens the often-stated argument that *D. suzukii* is characterised by a hard-wired adaptation to healthy fruits^3,4,18^.

Surprisingly, in Experiment 3, *D. suzukii* larvae had a higher developmental success in wounded than in healthy blueberries. However, the effects of intraspecific competition on two important fitness traits, body size and development time, were stronger in wounded than in healthy fruits. To date, we can only speculate about the mechanisms underlying this finding. Possibly, essential dietary microbes^17^ establish more likely and thrive better in the open flesh of wounded fruits, compared to the spatially very restricted self-created larval feeding galleries in healthy fruits. Because in preliminary experiments egg-to-adult survival in blueberries sterilized prior to oviposition does not seem to differ significantly from survival in unsterilized ones, transmission of beneficial microbes during oviposition may be critical for offspring performance. Drosophilids are assumed to exhibit an only primitive mode of offspring provisioning with microbes though^19^, and the association with dietary yeasts seems rather unspecific. These circumstances might constrain *D. suzukii* females in establishing optimal developmental conditions for their larvae and thus render healthy fruits not the ideal habitat. However, when individual larvae succeed in establishing a beneficial exo-microbiome in their feeding galleries they seem to suffer less from competition, compared to larvae feeding communally in the open flesh of wounded fruits. Field observations clearly demonstrate the ecological success of *D. suzukii* in healthy fruits and our lab-generated data on the fitness consequences should be interpreted with caution. Factors such as interspecific larval competition^20^ that might reduce *D. suzukii*’s success in wounded fruits need to be taken into account to fully understand the performance of this pest insect in the field.

## Conclusion

In a multi-host environment *D. suzukii*’s oviposition decisions are biased towards specialisation for healthy fruits, yet it possesses sufficient flexibility resulting in the use of wounded and fermenting fruits. *D. suzukii* can therefore be expected to readily accept and thrive in alternative substrates when the preferred healthy fruit stages are not available. When healthy host fruits are scarce, windfall fruit may thus constitute a critical reproductive refuge where this pest insect can persist at high population densities and from which it may re-colonise fruit plantations. This expectation is supported by a higher fitness return in wounded than in healthy fruits; however, this finding needs to be verified at field conditions. Nonetheless, a sustainable removal of windfall fruits might contribute to avoiding the establishment of locally stable and high-density *D. suzukii* populations in plantations^21^.

## Material and Methods

The *D. suzukii* population used in this study originated from ~50 flies that emerged from elderberries collected in September 2016 in northern Germany. Since then the flies had been kept at a population size of approx. 200 flies in custom-made population cages (22 litres). For egg collection, flies were offered thawed raspberries, and oviposited fruits were subsequently transferred to a standard *Drosophila* culture medium^22^. Larvae were incubated at 25 °C and emerging flies were released into a new population cage. Flies were fed with the decayed fruit/culture medium and crushed raspberries and had access to water.

Seven to ten-day old females, previously kept in the abovementioned population cages containing flies of the same age cohort were used in experiments 1 and 2. Prior to the experiments, every two days flies were given 15 to 20 thawed raspberries for oviposition. Thus, flies had no previous experience with blueberries, and competition for oviposition sites was expected to be intense. That is, compared to the situation in some treatments of the experiments, individual flies very likely did not experience egg limitation. In all experiments, the blueberries used were organically grown in Chile, carefully rinsed with water before further use and inspected for previous infestation and lesions.

### Experiment 1 – *Drosophila suzukii* egg distributions as a function of variation in total fruit availability

In a first experiment, we tested whether *D. suzukii* females have an oviposition preference for healthy host fruits (blueberries) given different degrees of resource availability. Resource availability was manipulated by offering individual female flies either 3, 9, 18 or 30 fruits. We assumed resource limitation to become more severe the fewer fruits were available. We did not use extraordinarily large and small fruits to prevent fruits size variation to have strong effects on the probability of fruit encounters and egg-laying decisions. Selected fruits were assigned randomly to the different treatments. To offer flies alternative fruit stages, fresh blueberries were wounded by cutting off the calyx and some of the surrounding fruit skin with a scalpel, exposing an area of exposed fruit flesh of ca. 1 cm diameter. Half of the wounded berries were left untreated, whereas the other half was inoculated with an aqueous yeast (*Saccharomyces cerevisiae*, strain DSM 70449) suspension (1 million yeast cells per 10 µl per fruit) to simulate a fermenting stage. Fruits were prepared approx. five hours prior to their use in the experiments. In each experimental arena, one third of the fruits offered was either ‘healthy’, ‘wounded’ or ‘fermenting’; e.g., when flies were offered 18 fruits in total, 6 were healthy, 6 wounded, and 6 fermenting. According to randomly assigned positions in a lattice, fruits were arranged in equal distances on a wet sponge cloth (cellulose/cotton blended fabric, Spontex, Germany) provided with holes to ensure that the wound remained on the upper side and to provide flies with water during the trials. The fruits were offered in 1 litre plastic boxes (arenas) (~6 × 11.5 × 17.5 cm, euroboxx, Germany) that were closed with a translucent lid. Around 4 p.m. single female *D. suzukii* females were released into the arenas. Initially, 15 arenas were prepared and incubated at a 16-hour light cycle and 25 °C ± 0.5. On the following day around 10 a.m. the flies were removed and the number of eggs per blueberry were counted. As we excluded counts were flies laid fewer than four eggs in total, the number of replicates was reduced accordingly (see Fig. 2).

The proportion of eggs single females allocated to the different fruit categories were used to test the null hypothesis that the status of the fruits – healthy, wounded or fermenting – had no influence on how the eggs were distributed, i.e. that on average 1/3 of the eggs laid were allocated to each fruit category. We explored this hypothesis by testing whether the proportion of eggs oviposited in healthy fruits changed as a function of total resource availability. This was achieved by applying a binomial generalised linear model (*GLM*) in R 3.3.2, for which we specified a *logit* link function. To test for an explicit deviation from 1/3 of eggs allocated to healthy fruits, we used the offset function to fix the intercept at *logit*(1/3); note that otherwise the null hypothesis is *logit*(1/2), which equals zero. For the data from experiment 2, the procedure was used to test for deviation from 1/2, 1/4, and 1/6, that is the expected relative abundance of eggs allocated to healthy fruit under the null hypothesis.

To explore the patterns of fruit use on the basis of presence/absence of eggs, i.e. proportion of fruits used as oviposition sites, we applied a generalised linear mixed model (*glmer*) in R with a *logit* link function. To test whether the proportion of fruits used for oviposition changed with total resource availability and as a function of the status of the fruits, we included the interaction term for resource availability (total number of fruits) and fruits status in the model.

### Experiment 2 – *Drosophila suzukii* egg distributions as a function of variation in the proportional abundance of healthy fruits

By manipulating the proportion of healthy fruits whilst keeping the total resource availability constant (12 blueberries), we aimed at testing whether the flies’ oviposition preference changes with the relative abundance of healthy fruits. This approach allows evaluating to what extent preference reflects specialisation on a certain type of host fruits^12^. Because in Experiment 1 wounded blueberries turned out to be the least preferred fruit category, we decided to reduce the proportion of healthy fruits embedded in a matrix of wounded blueberries by using following ratios of healthy/wounded fruits, respectively: 6/6, 4/8 and 2/10. The experimental procedure followed the same described in Experiment 1. Initially, 12 arenas were prepared, however, as we again excluded counts were flies laid fewer than four eggs in total, the number of replicates was reduced accordingly (see Fig. 4).

### Experiment 3 – Effect of host fruit status and intraspecific competition on the developmental success of *Drosophila suzukii* larvae

To obtain a range of egg densities on wounded and intact fruits, artificially wounded and healthy blueberries were exposed for differing durations to the flies in a population cage. Only egg densities of ≤20 eggs per gram were included in the analysis. In total, we obtained a range of egg densities in 111 healthy and 111 wounded fruits. After counting the eggs, berries were weighed and subsequently placed individually into 30 ml polystyrol rearing tubes (8 cm height, 3.5 cm diameter, K-TK, Germany) that were sealed with foam stoppers. The fruits were incubated at 25 °C ± 0.5, a 16-hour light cycle and ambient humidity in a thermostatic cabinet (Liebherr, Thermostatschrank, ET619-4/135 litre, Germany), and checked daily for emergence for a maximum of 25 days. Adults were frozen on the day of emergence, dried in a silica gel desiccator for eight days to constant weight and sexed under the microscope. For each blueberry, we weighed males and females separately (SE2 ultra-microbalance, Sartorius, Germany), and calculated the mean adult dry weight, i.e. the total weight of females, or males, divided by the number of flies measured for each sex. The developmental time was taken as the median number of days between oviposition (day 1) and the emergence of adult flies per blueberry.

We used generalised linear models (*GLM*s) to investigate the relation between the two fixed effects initial egg density (eggs per gram blueberry) and blueberry treatment (intact versus wounded fruits) and each response variable (survivorship, mean adult dry weight, median development time). As *Drosophila* is known to exhibit sexual size dimorphism^23^, sex was added as fixed effect in the analysis of adult dry weight. Development time as well as adult dry weight were modelled using a *GLM* with Gamma error structure and *log* link, whereas for survival data we specified a binomial *GLM* with a *logit* link function and corrected for under-dispersion. We performed stepwise backward eliminations of non-significant terms, starting with the most complex interactions (*Anova* function in *car* package^24^). Data were analysed in R 3.2.3^25^.

## Supplementary information

Supplementary Material.

Supplementary raw data

## Data Availability

All data generated and analysed during this study are included in this published article (and its Supplementary Information files)
