# Supplementary material for: Resource use by individual Drosophila suzukii reveals a flexible preference for oviposition into healthy fruits
Source: Sci Rep. 2020 Feb 21;10:3132. doi: 10.1038/s41598-020-59595-y (PMC7035383; doi:10.1038/s41598-020-59595-y)
Supplement: Supplementary file 1 — Supplementary Material. [file 41598_2020_59595_MOESM1_ESM.docx]

**Resource use by individual *Drosophila suzukii* reveals a flexible preference for oviposition into healthy fruits**

Renate Kienzle, Lara Groß, Shelby Caughman, Marko Rohlfs

| **Supplementary Table S1**. Experiment 1 – Generalised linear model (family = *quasipoisson*, link = *log*) output quantifying the effect of resource availability (3, 9, 18 or 30 fruits) on the total number of eggs laid by individual *D. suzukii* females. The estimates were used to draw the regression lines shown in Figure 1. | | | |
| --- | --- | --- | --- |
| **Factor** | **Estimate ±*s.e.*** | ***t*** | ***p*-value** |
| Intercept | 2.3482 ±0.2220 | 10.58 | <0.001 |
| log(no. fruits) | 0.3427 ±0.0799 | 4.29 | <0.001 |

| **Supplementary Table S2**. Experiment 1 – Generalised linear model (family = *quasibinomial*, link = *logit*) output quantifying the effect of resource availability (3, 9, 18 or 30 fruits) and the total number of eggs laid by individual *D. suzukii* females on the allocation of eggs to healthy fruits. | | | |
| --- | --- | --- | --- |
| **Factor** | **Estimate ±*s.e.*** | ***t*** | ***p*-value** |
| Intercept | 0.2620 ±0.4833 | 0.54 | 0.591 |
| Resource availability | -0.0138 ±0.0192 | -072 | 0.476 |
| Total eggs laid | 0.0053 ±0.0162 | 0.32 | 0.747 |

| **Supplementary Table S3**. Experiment 1 – Generalised linear mixed effect model (family = *binomial*, link = *logit*, ‘experimental arena’ as random factor) output quantifying the effect of resource availability (3, 9, 18 or 30 fruits) and fruit status (healthy, damaged fermenting) on the proportion of eggs deposited by *D. suzukii* females into these fruit categories. | | | |
| --- | --- | --- | --- |
| **Factor** | **Estimate ±*s.e.*** | ***z*** | ***p*-value** |
| Resource availability (RA) | 0.0326 ± 0.0214 | -1.52 | 0.128 |
| Fruit status - wounded | 0.2831 ±0.4772 | 0.59 | 0.553 |
| Fruit status - fermenting | 1.8941 ±0.5288 | 3.58 | <0.001 |
| Fruit status - healthy | 3.9363 ±0.7727 | 5.09 | <0.001 |
| RA x Fruit status - fermenting | -0.0620 ±0.0259 | -2.39 | 0.017 |
| RA x Fruit status - healthy | -0.0876 ±0.0320 | -2.74 | 0.006 |

| **Supplementary Table S4**. Experiment 1 – Generalised linear model (family = *quasibinomial*, link = *logit*) outputs quantifying the effect of resource availability on the proportion of blueberry fruits used by *D. suzukii* females for oviposition, separately, for either healthy, fermenting or wounded fruits. We ran these separate models based on the significant effect of the statistical interaction between ‘Resource availability’ and ‘Fruit status’ (see Results section of the main paper and Supplementary Table S3). | | | | |  |
| --- | --- | --- | --- | --- | --- |
| **Fruit status** | **Factor** | **Estimate ±*s.e.*** | ***t*** | ***p*-value** | |
| Healthy | Intercept | 3.4585 ±0.9706 | 3.56 | 0.001 | |
|  | Resource availability | -0.1037 ±0.0365 | -2.85 | 0.007 | |
| Fermenting | Intercept | 1.6077 ±0.4379 | 3.67 | <0.001 | |
|  | Resource availability | -0.0810 ±0.0184 | -4.40 | <0.001 | |
| Wounded | Intercept | 0.3216 ±0.5156 | 0.62 | 0.537 | |
|  | Resource availability | -0.0292 ± 0.0220 | -1.33 | 0.191 | |

| **Supplementary Table S5**. Experiment 2 – Generalised linear model (family = *quasibinomial*, link = *logit*) output quantifying the effect of the relative abundance of healthy fruits on the allocation of eggs by *D. suzukii*; see Figure 4. | | | |
| --- | --- | --- | --- |
| **Factor** | **Estimate ±*s.e.*** | ***t*** | ***p*-value** |
| Intercept | -2.536 ±0.408 | -6.216 | <0.001 |
| Rel. fruit abundance (healthy) | 6.682 ±1.165 | 5.736 | <0.001 |

| **Supplementary Table S6**. Experiment 3 – Minimum adequate generalised linear model (family = *gamma*, link = *log*) output quantifying the effect of egg density (eggs per mg fruit), fruits status (healthy, wounded), and sex (female, male) on the weight (mg) of emerged adult *D. suzukii* flies. Starting from a full model comprising all possible statistical interactions, backward elimination of non-significant terms was used. The estimates were used to draw the regression lines shown in Figure 5. | | | |
| --- | --- | --- | --- |
| **Factor** | **Estimate ±*s.e.*** | ***t*** | ***p*-value** |
| Intercept | -0.6827 ±0.0418 | -16.31 | <0.001 |
| Egg density | -0.0385 ±0.0036 | -10.58 | <0.001 |
| Fruit status (healthy) | -0.4065 ±0.0516 | -7.88 | <0.001 |
| Sex (males) | -0.2986 ±0.0264 | -11.30 | <0.001 |
| Fruit status x Egg density | 0.0260 ±0.0058 | 4.47 | <0.001 |

| **Supplementary Table S7**. Experiment 3 – ANOVA (Type II) quantifying the variation in *D. suzukii* fitness parameters explained by different factors. | | | | |
| --- | --- | --- | --- | --- |
| **Fitness parameters**  **(model type)** | **Factor** | ***F*** | ***d*.*f*.** | ***p* values** |
| Survival  (binomial *GLM*, *logit* link) | Fruit status | 6.262 | 1,220 | 0.013 |
|  |  |  |  |  |
| Adult dry weight  (gamma *GLM*, *log* link) | Sex  Egg density  Fruit status  Egg density x Fruit status | 126.502  97.333  53.946  20.258 | 1,309  1,309  1,309  1,309 | <0.001  <0.001  <0.001  <0.001 |
|  |  |  |  |  |
| Development time  (gamma *GLM*, *log* link) | Egg density  Fruits status  Egg density x Fruit status | 18.801  1.470  4.537 | 1,194  1,194  1,194 | <0.001  0.227  0.034 |
